# Disentangling the meat paradox: A comparative review of meat‐related conflicts across dietary behaviours

**DOI:** 10.1111/bjso.70062

**Published:** 2026-03-11

**Authors:** Benjamin Buttlar, Shiva Pauer

**Affiliations:** ^1^ University of Trier Trier Germany; ^2^ Helmut Schmidt University Hamburg Germany

**Keywords:** ambivalence, cognitive conflict, coping, dissonance, meat consumption, vegetarianism

## Abstract

A growing field of research examines how people experience and resolve cognitive conflicts in their behaviours, particularly in relation to meat consumption. Despite the alleged importance of conflict in behaviour change, most research focuses on how conflict motivates individuals to change or maintain their conflicted behaviour but disregards that conflict may persist even after successful behaviour change. This oversight has contributed to seemingly contradictory conclusions by conflating different kinds of conflicts and has arguably constrained theory development. Our review thus delineates (a) how people with different dietary patterns in meat consumption are affected by meat‐related ambivalence and dissonance, (b) differences in the characteristics (magnitude, frequency, moralization) of these conflicts, (c) boundary conditions of why conflict experiences arise, and (d) how these factors determine the downstream consequences of conflict. This allows us to derive several novel predictions, ranging from why conflict avoidance strategies may sometimes paradoxically increase the likelihood of experiencing conflict to the distinct roles of capability, opportunity, and motivation in shaping the behavioural consequences of conflict. By re‐evaluating prevailing assertions in the literature on meat‐related conflict, we offer numerous theoretical and practical implications regarding cognitive conflict and the psychology of meat consumption and avoidance.

Meat consumption has become a prominent case study for investigating how people experience and regulate cognitive conflict. This is because it exemplifies a paradigmatic approach to examining and understanding attitudinal ambivalence and cognitive dissonance (Bastian & Loughnan, 2017; Rozin, 2007). A whole line of psychological research investigates the antecedents, boundary conditions, and downstream consequences of meat‐related cognitive conflict (Gradidge et al., 2021; Rothgerber & Rosenfeld, 2021; van Gent et al., 2024). This research contributes to understanding why a majority of people can uphold their meat‐based diets despite conflict, while only a minority changes conflicted behaviour (Bastian & Loughnan, 2017; Buttlar et al., 2024; Rothgerber, 2020). Thereby, it provides a glimpse of how cognitive conflict is involved in decision‐making and the maintenance or change of (morally) troublesome behaviour more generally (Bastian, 2019; Feinberg et al., 2019). By examining the motivational and behavioural consequences of meat‐related conflict, the literature thus contributes to understanding and facilitating healthy and sustainable lifestyles that align with people's moral values.

Despite the alleged importance of conflict in behaviour change, much research focuses on how meat‐related conflict motivates people to change their behaviours rather than on how conflict may persist even after successfully changing a conflicted behaviour. For instance, the most influential theoretical models of meat‐related dissonance argue that by stopping to eat meat, people would resolve their conflict towards eating meat (e.g., Bastian & Loughnan, 2017; Rothgerber, 2020). However, not only people who eat meat (omnivores) but also those who eschew meat (vegetarians and vegans; from hereon veg*ans) can experience meat‐related conflict. This has been demonstrated in recent empirical research and theorizing on meat‐related ambivalence, which suggests that veg*ans can be torn between the positive and negative sides of eating meat and are, thus, prone to dietary lapses despite their more general commitment to eschew meat (Buttlar et al., 2023, 2024; Buttlar, Pauer, Scherrer, et al., 2025; Buttlar, Pauer, & van Harreveld, 2025).

In fact, people might switch back and forth in their diets, and many veg*ans have been found to have violated their diets (Rosenfeld, 2018; Ruby, 2012). A study of former and current veg*ans, for instance, indicated that 30% of former veg*ans and 16% of current veg*ans had already switched back and forth between a meat‐less and meat‐based diets (Asher & Green, 2016); and even current self‐identified vegetarians seem to eat meat from time to time as evidenced in a qualitative study, for example, in which 51% of participants from the United States indicated having eaten meat since the becoming a vegetarian for various reasons (e.g., craving for meat or social pressure to eat it or connecting meat to traditions and the desire to not being a burden at social events; Rosenfeld & Tomiyama, 2019). This suggests that conflict can be crucial to understanding not only how people change but also the processes and outcomes in efforts to maintain the dietary change. Despite this, there is no integrative theoretical framework that provides a holistic understanding of who experiences meat‐related conflict, under which conditions it arises, and what its experiential characteristics and potential consequences are.

In this review, we will thus review the literature on meat‐related ambivalence and dissonance based on four key questions: Which groups of people experience which kinds of meat‐related cognitive conflicts? Why do they experience these conflicts? When do they experience it? And what are the motivational and behavioural consequences? To do so, we distinguish between attitudinal ambivalence and cognitive dissonance – two distinct yet related psychological states that differ in their structure: While ambivalence represents a *conflict within an attitude*, dissonance represents a conflict between a *commitment and an attitude* (Buttlar, Pauer, & van Harreveld, 2025). While previous work examines this distinction and interrelations between ambivalence and dissonance within decision‐making (Buttlar, Pauer, & van Harreveld, 2025), we will outline that both people who eat meat and those who abstain from it experience ambivalence and dissonance, depending on the attitudes and commitments involved in their dietary behaviours. This, in turn, allows us to examine how people experience these conflicts as a function of the underlying components, how they might avoid conflict, and how they differentially cope with it when they experience it nonetheless.

## WHO EXPERIENCES MEAT‐RELATED CONFLICT?

Research on meat‐related conflict provides insightful perspectives on conflict dynamics and behaviour change because the topic involves different dietary groups with polarized attitudes and distinct behavioural commitments (e.g., Barr & Chapman, 2002; Buttlar et al., 2024; Povey et al., 2001; Rosenfeld & Burrow, 2018): People who eat meat (i.e., omnivores) typically hold predominantly positive attitudes about it, whereas those who abstain from meat hold predominantly negative attitudes about it (i.e., veg*ans; Buttlar et al., 2023). To disentangle meat‐related conflicts, we therefore distinguish between conflicts that arise due to ambivalent attitudes as opposed to counterattitudinal behaviour, as well as their differences across dietary groups.

Ambivalent attitudes involve inconsistent positive and negative thoughts and affections (herinafter evaluations), called potential (or objective) ambivalence. Potential ambivalence can result in a discomforting meta‐cognitive conflict about the evaluative inconsistency called felt (or subjective) ambivalence (Priester & Petty, 1996; van Harreveld et al., 2015). Both omnivores and veg*ans report being ambivalent about meat (Buttlar et al., 2023; Buttlar & Walther, 2018). In fact, 94.3% of omnivores and 70.8% of veg*ans among a sample of German students reported at least one evaluation that conflicted with their predominant attitude (Buttlar et al., 2023). Going beyond these evaluations, a total of 66.8% of meat eaters in a representative sample of the German public have also reported feeling conflicted about meat consumption (Pauer et al., 2022). These findings are corroborated by research using self‐report and behavioural process‐tracing methods, showing that veg*ans can also experience ambivalence about eating meat, albeit less so than omnivores (Buttlar et al., 2023; Buttlar & Walther, 2018).

Dissonance arises when people hold inconsistent cognitions, commonly involving a conflict between a commitment to behaviour and an attitude or a belief (Brehm & Cohen, 1962; Cooper & Fazio, 1984; Festinger, 1957, 1964). We propose that both omnivores and veg*ans experience meat‐related dissonance, such as when they commit to eating meat while evaluating it negatively. Commitments can be thought of as the expectancy to continue with a chosen action (Janis & Mann, 1977). People can have different degrees of commitment, which are determined by the irrevocability, explicitness, voluntariness, importance, and frequency of a given decision (Kiesler & Sakumura, 1966). In this vein, research on meat‐related dissonance shows that omnivores experience dissonance when they eat meat or anticipate doing so (Bastian et al., 2012; Jacobs et al., 2024; Loughnan et al., 2010); omnivores also seem to experience dissonance merely when their dietary commitment becomes accessible due to thinking of a veg*an (Rothgerber, 2014). It has been argued that dietary change to veg*anism resolves these conflicts, such that veg*ans would not experience meat‐related dissonance anymore (Bastian & Loughnan, 2017; Rothgerber, 2020). However, many veg*ans violate their diets from time to time by eating meat (Rosenfeld & Tomiyama, 2019; Ruby, 2012; Williams et al., 2023). Thus, we argue that these lapses elicit dissonance due to a situational commitment to a meat‐based food choice that conflicts with their predominantly negative attitude.

In summary, we extend prevailing frameworks on meat‐related conflict (Bastian & Loughnan, 2017; Piazza et al., 2023; Rothgerber, 2020) by suggesting that both omnivores and veg*ans experience meat‐related conflict in the form of ambivalence and dissonance. Disentangling these meat‐related conflicts across dietary groups illuminates more comprehensive conflict dynamics and thereby advances the understanding of why and when people experience conflict and how it affects the ways in which they cope with conflict.

## WHY DO PEOPLE EXPERIENCE MEAT‐RELATED CONFLICT?

A fundamental need for cognitive consistency makes experiences of inconsistencies, like ambivalence and dissonance, unpleasant (Festinger, 1957; McGregor et al., 2019; van Harreveld, van der Pligt, et al., 2009). Ambivalence and dissonance both conceptualize why cognitive conflicts emerge and how people resolve them, with a number of shared antecedents and consequences (Buttlar, Pauer, & van Harreveld, 2025; Proulx et al., 2012). Despite their similarity and the fact that both types of conflict are partly rooted in people's attitudes, they arguably constitute distinct states with different antecedents and elicitors. These differences are crucial for conceptualizing the experiences of these conflicts and their downstream consequences in the subsequent sections.

Many people evaluate meat positively and negatively at the same time for a variety of reasons (Berndsen & van der Pligt, 2004; Buttlar et al., 2023; Pauer et al., 2022; Ruby et al., 2016): People can evaluate meat positively because they cherish meat's nutritional density, taste, and associated cultural traditions, while they can simultaneously evaluate it negatively due to the detrimental consequences of meat concerning animal suffering, human health risks, and environmental damage. Together, these opposing evaluations are the basis of potential and felt ambivalence in omnivores as well as veg*ans (Buttlar et al., 2023, 2024; Pauer et al., 2022). Importantly, the evaluative structure underlying these attitudes differs between omnivores and veg*ans regarding their valence, domains, and moral appraisals. The two groups hold distinct predominant attitudes towards meat (Barr & Chapman, 2002; Buttlar & Walther, 2018): Omnivores evaluate meat predominantly referring to positive evaluations, while veg*ans' evaluations involve mainly negative ones. As such, omnivores experience felt ambivalence when negative evaluations conflict with their predominantly positive attitudes, while veg*ans experience felt ambivalence, especially when positive evaluations arise in conflict with their predominantly negative attitudes (Buttlar et al., 2023).

Eating meat is also considered a typical behaviour that elicits cognitive dissonance if the commitment to eating meat is inconsistent with an individual's deeply held attitudes (Bastian & Loughnan, 2017). Most people eat meat despite believing that it causes harm to animals, the environment, or personal health (Gradidge et al., 2021; Hartmann & Siegrist, 2020; Rothgerber & Rosenfeld, 2021). The resulting cognitive dissonance that people perceive when their commitment to eating meat contradicts a positive, coherent, and moral self‐image has thus been termed the meat paradox (Bastian & Loughnan, 2017; Loughnan et al., 2010; Rothgerber, 2020). The literature on the meat paradox suggests that people experience dissonance when they become aware of a conflict between their commitment to eating it and their negative attitude about meat. Although research on meat‐related dissonance has mainly focused on conflicts about eating meat (Gradidge et al., 2021), we argue that dissonance could arise for similar reasons when people commit to eschewing meat from their diet despite holding contradictory positive evaluations of meat – such as in terms of its taste, sociability, or nutritiousness – as long as their commitment stands in contrast to (self‐)relevant attitudes. As such, meat‐related dissonance could not only arise when people decide to eat meat but also in people who decide to follow a diet *without* meat because the resulting commitment can still conflict with one part of their attitude.

### Conflict strength

The constituents underlying conflict may influence how intensely it is experienced. In eliciting felt ambivalence, negative evaluations have more weight than positive evaluations (i.e., negativity bias and positivity offset; Cacioppo et al., 1997; Snyder & Tormala, 2017). Thus, when omnivores who hold predominantly positive attitudes towards meat also have even minor contradictory negative evaluations about it, these may already elicit intense felt ambivalence; to the contrary, when veg*ans (with a predominantly negative attitude) have the same amount of contradictory (i.e., positive) evaluations, these arguably lead to less intense felt ambivalence.

The same logic could hold for the intensity of dissonance. As conflicting positive evaluations are less potent than conflicting negative evaluations, we argue that a commitment to eschew meat might result in intense dissonance (when positive evaluations arise) compared with a commitment to eat meat (when negative evaluations arise). Nonetheless, attitudinal differences between dietary groups may influence how exactly dissonance is experienced when people situationally commit to eat or eschew meat: When people decide to eat meat, veg*ans who situationally commit to eating meat may experience dissonance more strongly than omnivores because they have more negative evaluations and these evaluations are more influential compared with the positive ones; when people decide to eschew meat, however, omnivores might experience more intense dissonance because they carry more positive evaluations towards meat compared with veg*ans even if those evaluations are less potent.

Moreover, we propose that people experience more intense conflict to the degree to which it involves a moralized attitude. Moralization involves adopting a firm position on an issue and viewing one's stance as grounded in judgements of right and wrong (Feinberg et al., 2019; Rozin, 1999). This process often begins with a moral shock, which occurs when exposure to new information reveals that something once seen as neutral actually causes harm (Wisneski & Skitka, 2017). Push factors like moral emotions and moral cognitions foster moralization, while pull factors like hedonic motivations and rationalizations can reduce it (Feinberg et al., 2019). Depending on the outcome of this moralization process, people see their attitudes more or less strongly based on their beliefs of what is ‘right’ and ‘wrong’ (Skitka et al., 2021). When attitudes are moralized, conflict may elicit more intense emotions, like feelings of regret because of failing to act upon moral convictions (Skitka et al., 2017), and heightened physiological arousal (Garrett, 2019). Given that these affective states are central to the experience of cognitive conflict (Harmon‐Jones et al., 2025; McGrath, 2017; van Harreveld, van der Pligt, et al., 2009), we argue that moralized attitudes amplify the intensity of conflict. Crucially, veg*ans already possess more moralized attitudes towards meat compared with omnivores. These attitudes often include evaluations tied to people's sense of morality, for example, concerning animal welfare or environmental issues (Finkhäuser et al., 2025; Rosenfeld, 2019; Rozin et al., 1997). As such, experiences of ambivalence and dissonance might be more intense if people's attitudes about meat are tied to their core beliefs of what is right and what is wrong. As this moralization is especially pronounced in veg*ans, conflicted veg*ans might experience a more pronounced intensity of conflict compared with conflicted omnivores.

## WHEN DO PEOPLE (NOT) EXPERIENCE MEAT‐RELATED CONFLICT?

People experience felt ambivalence or cognitive dissonance when the underlying inconsistent components become simultaneously accessible (McGregor et al., 2019; Newby‐Clark et al., 2002). This key role of accessibility may involve reflecting on inconsistent components, for instance, in decision situations (Buttlar, Pauer, & van Harreveld, 2025; Festinger, 1964; van Harreveld, van der Pligt, et al., 2009).

Felt ambivalence about meat arises when an ambivalent attitude becomes accessible and when people need to make decisions based on it. Pauer et al. (2022) showed, for instance, that omnivores' felt ambivalence increased when they were asked to recall personally held positive and negative evaluations of meat. In addition, daily fluctuations in potential ambivalence about meat elicited felt ambivalence over time among omnivores in a recent experience sampling study, especially during moments of food choice when people felt they had to decide what to eat and elaborate on it (Pauer et al., 2024). Similarly, a recent diary study among vegetarians demonstrated that felt ambivalence arises more strongly when both positive and negative evaluations about meat become accessible (Buttlar, Pauer, Scherrer, et al., 2025). In addition to these everyday‐life studies, laboratory research has used the mouse‐tracking methodology, showing that many omnivores' and veg*ans' are literally torn in their movements when deciding whether meat dishes are positive or negative (Buttlar & Walther, 2018, 2022). These findings support the notion that felt ambivalence emerges especially when people have to make a choice, as decision‐making draws attention to an ambivalent attitude for committing to one side of it (van Harreveld, Rutjens, et al., 2009; van Harreveld, van der Pligt, et al., 2009).

After committing to attitude‐inconsistent food choices about meat, people experience meat‐related dissonance (Pauer et al., 2024). In early studies on meat‐related dissonance, omnivores were, for instance, prompted to eat meat, or they anticipated doing so later in the study (Bastian et al., 2012; Loughnan et al., 2010). Going beyond, dissonance was induced in omnivores by reminding them of their long‐term commitment to eating meat, for example, by using vignettes about vegetarians (Rothgerber, 2014). While earlier work has argued that dissonance rarely arises in daily life due to preventive conflict avoidance strategies (e.g., Bastian & Loughnan, 2017), more recent experience sampling studies have shown that meat‐related conflicts can arise in daily life, including regular experiences of dissonance in omnivores (Buttlar, Pauer, Scherrer, et al., 2025; Pauer et al., 2024). Based on these studies, it becomes apparent that various situations can make the inconsistency between meat‐related commitments and attitudes accessible and thus elicit dissonance, even with regular recurrence of the conflict.

### Conflict recurrence

Crucially, omnivores seem to experience conflict more often than veg*ans (Buttlar et al., 2023), and as with conflict intensity, we argue that this lies in the fact of how conflict is constituted. Based on the premise that conflict is more potent if the conflicting valence is negative, people holding few negative but many positive evaluations (like omnivores towards meat) towards an attitude object may thus not only experience more intense conflict but may be more prone to experiencing felt ambivalence compared with others (like veg*ans) who hold few positive and many negative evaluations (Snyder & Tormala, 2017). This is because the accessibility of relatively minor negative (but not positive) evaluations about eating meat may elicit conflict if it conflicts with a predominantly positive attitude or a commitment to eating meat. As such, it simply takes less that omnivores experience meat‐related conflict, which explains why omnivores experience meat‐related conflict more often than veg*ans.

We also argue that the factors contributing to attitude moralization may affect how frequently people experience conflict. However, in contrast to the more general effect of attitude moralization on conflict intensity, the push and pull factors, which determine the outcome of the moralization process, make people prone to experiencing meat‐related conflict. In particular, we suggest that omnivores become more prone to experiencing conflict when push factors towards moralization fuel the moralization process, and that veg*ans are more prone to experiencing conflict when encountering pull factors away from moralization (Finkhäuser et al., 2025). Specifically, we argue that the accessibility of push factors towards moralization might render an attitude as more moralized and thereby make it more likely that omnivores experience conflict (adding to the negative valence of such associations with eating meat); in contrast, the accessibility of pull factors may make the predominant (negative) attitude of veg*ans more pliable because less moralized attitudes are less stable (Luttrell & Togans, 2021), making conflict more likely. To this end, a qualitative study shows that omnivores most frequently mention (negative) push factors tied to animal ethics as reasons why they feel ambivalent, while veg*ans most often report reasons based on pull factors concerning the positive social or sensory implications of eating meat (Buttlar et al., 2023). These qualitative findings for omnivores are supported by an experimental study, which showed that omnivores experience dissonance about eating meat due to a text involving push factors towards moralization on the environmental or animal welfare issues associated with meat that highlight its moral implications (but not due to associations with personal health issues; Silva Souza & O'Dwyer, 2022).

### Conflict avoidance

Given that ambivalence and dissonance are temporary states that arise only if their inconsistent underpinnings become accessible (Pauer et al., 2024), they can remain dormant even in decision situations (Bastian & Loughnan, 2017). In fact, the literature on meat‐related dissonance suggests that people avoid experiencing conflict before it even occurs by using at least five conflict avoidance strategies (see Bastian & Loughnan, 2017; Rothgerber, 2020): wilful ignorance (Leach et al., 2022; Onwezen & van der Weele, 2016), the dissociation of meat from its animal origin (Benningstad & Kunst, 2020; Kunst & Hohle, 2016), the formation of habits (Rees et al., 2018; Saba & Di Natale, 1998), the spread of social norms (Kawamura & Kusumi, 2020; Lea & Worsley, 2001), as well as ritualization and institutionalization (Bastian & Loughnan, 2017). These preemptive strategies arguably enable people to engage in attitude‐inconsistent behaviour without experiencing dissonance. Going beyond, however, we argue that they may also prevent ambivalence before people commit to eating or eschewing meat.

Specifically, we posit that conflict avoidance strategies allow people to circumvent reflecting on inconsistencies, helping people to make and maintain an effective course of decision‐making and action. However, some conflict avoidance strategies might not function unanimously for everyone and sometimes even foster conflict. This becomes apparent when comparing dietary groups. We propose that individual conflict avoidance strategies, such as habit formation, might help avoid conflict for omnivores and veg*ans alike, whereas societal conflict avoidance strategies could have diverging effects. For instance, in most societies, meat consumption is a social norm – being reinforced by rituals (such as traditional dietary expectations for Christmas dinner) and institutional policies (such as subsidies for animal agriculture) that facilitate and encourage eating meat (Bastian & Loughnan, 2017). Although societal conflict avoidance strategies might help omnivores avert conflicts by strengthening the accessibility of positive aspects of eating meat while reducing the accessibility of negative aspects of eating it, the heightened accessibility of positive aspects may make veg*ans prone to experience ambivalence about eating meat. For instance, when veg*ans are confronted with a pro‐meat norm, this might increase the accessibility of positive attitudes, such as the nice taste or the sociability associated with a traditional Christmas dish.

However, societal conflict avoidance strategies are out of individual control and might be shifting and even reversing. In fact, current societal trends towards pro‐vegetarian norms (e.g., Carfora et al., 2022), for example, could lead to reduced ambivalence in veg*ans because they might render positive attitudes less accessible; yet, such trends may counteract existing conflict avoidance strategies in omnivores and thereby facilitate conflict experiences as they might increase the accessibility of the negative attitudes towards meat. In this way, contextualizing meat‐related conflict as a function of different dietary groups and their levels of accessibility of inconsistency fosters our understanding of conflict avoidance strategies, revealing the conditions under which they not only avert but also promote cognitive conflicts.

## WHAT ARE THE CONSEQUENCES OF EXPERIENCING MEAT‐RELATED CONFLICT?

Felt ambivalence and dissonance inflict aversive states (Elliot & Devine, 1994; van Harreveld, Rutjens, et al., 2009). Before a decision, ambivalence is typically associated with negative emotions such as anticipated regret or uncertainty (Itzchakov & van Harreveld, 2018; van Harreveld, Rutjens, et al., 2009), and after a decision, dissonance is typically associated with negative emotions, such as regret, guilt, anger, or shame (Harmon‐Jones et al., 2025; McGrath, 2017). To regulate this unpleasant experience, felt ambivalence, and dissonance motivate people to resolve the aversive state (Festinger, 1957; van Harreveld et al., 2015). Such coping strategies either aim at mitigating the conflict‐induced discomfort through emotion‐focused coping (e.g., diffusion of responsibility for meat consumption; Rothgerber et al., 2022) or at resolving the underlying conflict through problem‐focused coping, such as information‐seeking (Buttlar, Lambrich, McCaughey, et al., 2025; van Harreveld, van der Pligt, et al., 2009). Crucially, people do not necessarily invest effort in systematic problem‐focused coping, which aims at making the best decision, but instead often engage in biased problem‐focused coping; the latter aligns with the predominant attitude or commitment, and it has been argued that it is driven by insufficient capacity and motivation to engage with the topic through more systematic problem‐focused coping (Brehm, 1960; Nordgren et al., 2006; van Harreveld, van der Pligt, et al., 2009).

Building on the COM‐B framework (Bryant et al., 2023; Grassian, 2020; Michie et al., 2011), we additionally argue that opportunity (O) is a critical factor that determines people's coping effort, besides capability (C) and motivation (M). Lacking opportunity can undermine systematic problem‐focused coping, leading to biased problem‐ or emotion‐focused coping and ultimately preventing behaviour change (B). That is, even if people are capable and motivated to invest effort and cope with conflict more systematically, behaviour change also requires a perceived opportunity to do so.

### Capability

Capability refers to people's ability to perform a certain action (Michie et al., 2011). This encompasses both physical capability, such as for cooking after a workday, and cognitive resources, such as knowledge, mental skills, or psychological endurance required to undertake the behaviour. We argue that if people do not see themselves as capable of changing meat‐related behaviour, they will be more likely to engage in biased problem focused or emotion‐focused coping, aligning with their typical dietary behaviours. For instance, omnivores who are conflicted but lack knowledge for cooking meat‐free dishes will be less open to process information on meatless and meat‐based recipes alike; the same applies for veg*ans who are conflicted but do not know how to cook meat‐based dishes. Instead, people will tend to seek information that accommodates their pre‐existing dietary choices, as this requires less effort. This, in turn, hampers behaviour change.

Crucially, the capability to engage in behaviour change is usually higher when people experience ambivalence in comparison to dissonance. When people experience ambivalence before a decision, they sit on the fence with heightened perceptions of having a choice (Pauer et al., 2024), while tending to be more open to all available outcomes of the decision; once people experience dissonance, however, they have already committed to a decision, which eliminates non‐chosen options (unless the decision is reversible) and thereby reduces perceived capability (Buttlar, Pauer, & van Harreveld, 2025). For instance, before ordering in a restaurant, there are various options to choose from; however, after telling the waiter the order, it steadily becomes more difficult to change course, and the capability to behave in a different way fades. In this vein, it has been shown that omnivores who believe that choosing meatless alternatives would help them to reduce ambivalence indeed seek out more supportive information and show increased intentions to eschew meat (Pauer et al., 2022). Similarly, veg*ans are more likely to seek information about meatless options when feeling ambivalent about eating meat (Buttlar et al., 2023). By sparking systematic problem‐focused coping, ambivalence might thus not only lead to behavioural change in a given decision but also facilitate a heightened perception of capability, with downstream benefits for future decisions. By the same token, given the decreased capability *after* eating meat, dissonance increases the likelihood of engaging in biased problem‐focused or emotion‐focused coping that allows people to uphold their decisions, thereby ultimately preventing behaviour change. To this end, research has shown that prominent coping strategies with meat‐related dissonance include the denial of animals' minds and emotions (e.g., Bastian et al., 2012), the rationalization of meat consumption (Hopwood et al., 2021; Piazza et al., 2015), and the denial of responsibility for food choices (Graça et al., 2016; Pauer et al., 2024; Rothgerber, 2014). These studies have mostly investigated omnivores, but similar processes have been observed in lapsing veg*ans (e.g., Rosenfeld & Tomiyama, 2019).

### Motivation

Motivation represents the internal processes that guide and energize behaviour (Michie et al., 2011). It consists of reflective and automatic processes that shape behaviour: Reflective motivation involves conscious planning and evaluation (e.g., intentions to stop eating meat for ethical reasons), whereas automatic motivation involves non‐conscious responses (e.g., craving meat). Motivation can thereby facilitate or impede behaviour change. For instance, if people feel motivated to eat less meat due to ethical reasons, they might be more inclined to systematically seek information that could help change their behaviour compared with people who do not have such intentions.

People's motivation to engage in more effortful coping and thereby behaviour change may be affected by the type of conflict that people experience. In particular, dissonance makes people less motivated to change their behaviour (Buttlar, Pauer, & van Harreveld, 2025), arguably because they aim to act efficiently upon their decisions (Harmon‐Jones et al., 2009). When dealing with ambivalence, people are also sometimes inclined to act in line with their predominant attitude (e.g., Nordgren et al., 2006); however, compared with dissonance, there seems to be less selective motivation. In fact, ambivalence can even facilitate better decision‐making while reducing biases, such as the tendency to confirm pre‐existing attitudes and beliefs (Hohnsbehn et al., 2022). This is not to say that dissonance always leads to biased problem‐focused or emotion‐focused coping and ambivalence to systematic problem‐focused coping, respectively. As with ambivalence, we propose that people can sometimes feel motivated to cope with dissonance by using effortful, systematic problem‐focused coping and thus ultimately reconsider their behavioural commitments that elicited the dissonance. Here, we argue that conflict strength and recurrence foster people's motivation to engage in effortful coping, with central roles of the underlying attitude valence (i.e., conflicting negativity or positivity) and attitude moralization (see sections on conflict strength and conflict recurrence).

The magnitude of experienced conflict may affect how motivated people are to cope with it. In particular, we argue that intensely experienced conflict can motivate people to cope systematically with conflict and, in turn, decrease the likelihood that they side with their predominant attitude via emotion‐ or biased problem‐focused coping. This is because people are increasingly motivated to reduce conflict as its intensity rises. To this end, research has shown that the more ambivalent omnivores are, the more open they tend to be to forgoing meat; and people whose ambivalence is made salient aim to reduce it by seeking information on meat avoidance and behavioural change (Pauer et al., 2022). Similarly, when people are made aware of their dissonance, they can become more willing to reduce their consumption of animal products when the dissonance is due to a moralized text on environmental or animal welfare issues associated with meat (Silva Souza & O'Dwyer, 2022). The latter study even showed that people's attitudes about eschewing meat became more positive, reflecting attitude change in the opposite direction of their commitment.

The frequency with which people experience conflict may be another motivational determinant of coping styles. Felt ambivalence about meat consumption can persist over months or even longer (Finkhäuser et al., 2025) and is frequently re‐experienced in daily life (Pauer et al., 2024). Such recurrence of meat‐related ambivalence motivates people to establish a consistent attitude to avert the aversive recurrence of the ambivalence, for which they engage in more systematic problem‐focused coping (Pauer et al., 2022, 2023). More specifically, people seek information on meat avoidance, arguably to facilitate attitude change; and eventually, such systematic problem‐focused coping may indeed lead to more univalent attitudes (Buttlar, Pauer, & van Harreveld, 2025; Pauer et al., 2022). While the aforementioned literature suggests that repeated experiences of ambivalence affect people's motivation to cope more systematically, we propose that similar processes may influence how omnivores and veg*ans cope with dissonance. Initial support for this assumption comes from a recent experience sampling study on meat‐related conflict by Pauer et al. (2024) who traced the dynamics of omnivores' conflict in daily life and showed that dissonance initially leads omnivores to deny responsibility for choosing to eat meat, which concurs with alleviating the experienced conflict; crucially, when the conflict resurges afterward, people do not attempt to resolve the conflict through denying responsibility once again but, instead, tend to engage in rather problem‐focused coping, that is, evaluative spreading towards greater attitudinal consistency, suggesting attitudinal change. We therefore conclude that when initial coping fails to avert dissonance from recurring, this could motivate omnivores and veg*ans to engage in more effortful coping over time, albeit to different extents as mentioned before.

### Opportunity

In addition to capability and motivation – as factors situated within individuals – we argue that contextual variables that affect the perceived opportunity to act in a certain way play important roles in shaping people's coping attempts. Opportunity denotes the external factors that make the behaviour possible or facilitate its enactment (Michie et al., 2011). This includes the physical opportunity shaped by environmental conditions, ranging from individuals' available time and resources to the availability and physical cues provided by the environment and political frameworks. For instance, individuals' decisions depend on the availability of meatless dishes in a restaurant and the underlying subsidy for animal products (Hofmann, 2024). We argue that if people feel they cannot choose between meat‐based and meatless options, they will be less likely to engage in systematic problem‐focused coping when feeling conflicted. Such a perceived lack of choice will facilitate emotion‐ and bias‐problem‐focused coping towards the available option. This way, even if people are capable of and motivated to change a behaviour, low opportunity can counteract the tendency for systematic coping, leading them to make decisions aligned with environmental affordances.

Crucially, environmental factors do not only include physical opportunity but also social opportunity. Social opportunity arises from interpersonal and cultural influences, including social norms, cues, and language that affect actual and perceived behavioural expressions. For instance, perceived social stigma against vegetarians and vegans is an important barrier for many omnivores in adopting a meatless diet (Markowski & Roxburgh, 2019). And even after abandoning meat from the diet, veg*ans sometimes compromise and eat meat due to social pressure from pro‐meat norms (Salmivaara et al., 2022) or self‐silence and avoid expressing their dietary identity, such as by refraining from openly signing a petition for more plant‐based alternatives in the local cafeteria if this reflects the majority position (Bolderdijk & Cornelissen, 2022). As with physical opportunity, we argue that people will be less likely to engage in systematic problem‐focused coping in such situations because they afford individuals a limited set of choice options. For example, in the absence and persecution of meat options in vegetarian‐dominated villages in India, individuals might rarely face situations in which systematic problem‐focused coping provides any viable benefits but implies social deviance from traditional Indian consumption practices (Khara et al., 2021). By the same token, cultural changes towards less reliance on animal proteins but more plant‐based options in supermarkets and restaurants expose individuals to a choice and reflecting on why they would choose meat despite its downside, increasing the likelihood of engaging in problem‐focused coping. People might thus engage in more emotion‐ and biased problem‐focused coping when perceiving less physical and social opportunity.

## DISCUSSION

**FIGURE 1:**
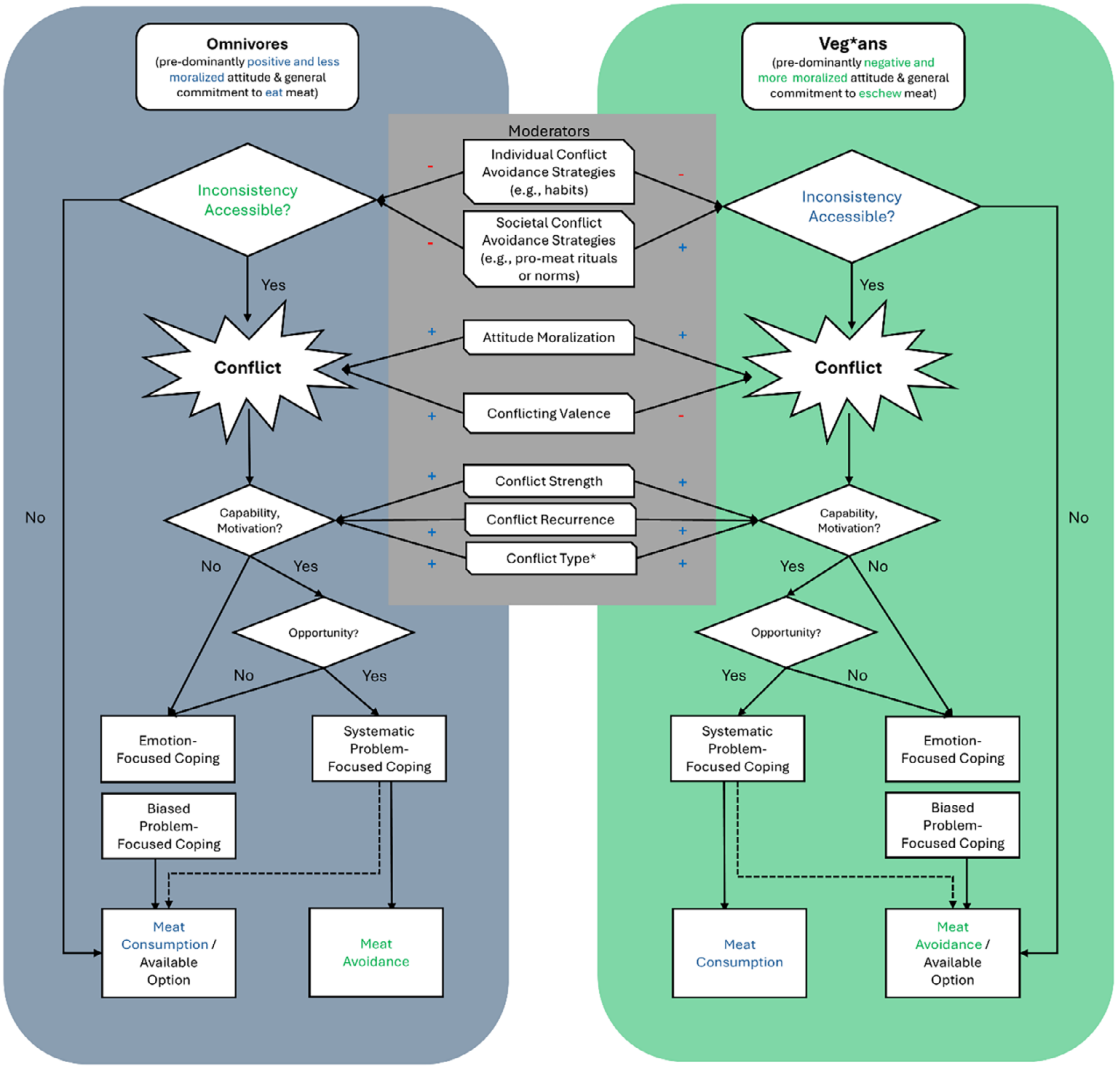
Schematic depiction of the similarities and differences the experience of meat‐related conflict between omnivores in veg*ans. For simplicity, the flow chart shows capability and motivation preceding opportunity; however, these internal and external factors could also be considered in reverse order and might even affect each other (see Bryant et al., 2023; Michie et al., 2011). The dashed line indicates that, while systematic problem‐focused coping may lead to behaviour in line with one's predominant attitude or previous commitments, that outcome becomes less likely once people engage in systematic problem‐focused coping compared with emotion‐ or biased problem‐focused coping.

In this review, we re‐examined and integrated the literature on meat‐related cognitive conflict. Within this field, we argue that the distinction between people who eat meat and those who abstain from it has so far been neglected, hampering theory development and overlooking crucial implications for understanding how cognitive conflict contributes to behaviour change and maintenance. Through our review, we thus aim to clarify how omnivores and veg*ans experience meat‐related ambivalence and dissonance. By giving a systematic overview of this up‐and‐coming field of research (Figure 1), we provide novel insights into the processes involved in the experiences, boundary conditions, and consequences of meat‐related conflict. Thereby, we hope to uncover new areas of research that further our understanding of the multifaceted roles of cognitive conflict in behavioural change and maintenance.

By identifying who experiences meat‐related conflict, we show that both omnivores and veg*ans experience conflict, albeit with different underlying attitude structures and commitments towards eating meat. This holds true for ambivalence and dissonance, contrary to the assumptions raised by other models on meat‐related dissonance. These models operate on the assumption that veg*ans experience no meat‐related conflict, as they presumably lack a commitment to eating meat (Bastian & Loughnan, 2017; Piazza et al., 2023; Rothgerber, 2020). Although veg*ans are not committed to following a meat‐based diet by definition (Ruby, 2012), they occasionally violate their diets (Rosenfeld & Tomiyama, 2019) and thereby commit to eating meat. As such, when veg*ans eat meat, their commitment to eat meat leads to dissonance in a given situation because it is inconsistent with their attitudes and beliefs. Empirical research should, therefore, compare the mechanisms involved in how omnivores and veg*ans either maintain or change their diets in the face of conflict.

As our analysis differentiates these dietary groups, we highlight the crucial roles of the distinct attitudinal origins of meat‐related conflicts in explaining why people experience ambivalence and dissonance. That is, the valence of the conflicting attitudinal component (as opposed to the predominant component) underlying an inconsistency and attitude moralization constitutes boundary conditions that shape how people experience the conflict. In particular, we argue that people experience more pronounced meat‐related conflict when the conflicting evaluative components with a negative valence increase or become accessible (Snyder & Tormala, 2017). As such, heightened negativity about meat is particularly likely to elicit conflict in people who hold a predominantly positive attitude or a commitment to eating it, like omnivores, because this attitude structure can elicit experiences of conflict even if its magnitude is relatively small (as compared with people with a predominantly negative attitude, like veg*ans). Relatedly, we highlight that conflict is experienced more strongly if an attitude is moralized, especially because contemplating moralized attitudes elicits physiological arousal (Garrett, 2019) and acting against them leads to regret (Skitka et al., 2017). This perspective is crucial for understanding why eating meat is a particularly potent elicitor of meat‐related conflict. Nonetheless, our analysis also implies that people can experience dissonance when they commit to eschewing meat if it conflicts with positive evaluations of meat. This may particularly be the case if the commitment is inconsistent with self‐relevant positive sides of eating meat. Thus, we propose that the field needs to move beyond its current focus on the negative valence underpinning conflict about eating meat to provide a more holistic perspective on the attitudinal origins of meat‐related conflict and how it affects conflict experiences across dietary groups.

Crucially, we posit that ambivalent and dissonant conflict arise when the inconsistent cognitions underlying it become accessible (see also McGregor et al., 2019; van Harreveld et al., 2015), that is, when they come to people's minds. While previous accounts have already suggested that meat‐related dissonance can be avoided by blocking its triggers (Bastian & Loughnan, 2017; Rothgerber, 2020), the present review sheds light on the mechanisms that underlie such conflict avoidance strategies and explains how they help to prevent conflicts before they unfold. That is, omnivores may avoid conflict, for instance, in social contexts with a predominant pro‐meat norm because these norms make the negative attitude components less accessible. By explicating these mechanisms, we also show, however, that the same norms may increase the likelihood that veg*ans experience conflict because such norms may also make the positive aspects of meat more accessible. Of course, social contexts are variable and a pro‐meat norm might be present in one context but not in others, or it might change over time, which may determine how people experience meat‐related conflict. By outlining the processes that make people experience conflict, our analysis thus offers fruitful implications about the boundary conditions that determine when people experience conflict, underscoring the differential effects of societal conflict avoidance strategies for omnivores and veg*ans.

Finally, our review of the downstream consequences of meat‐related conflicts highlights why ambivalence and dissonance play different roles in behaviour change: capability, opportunity, and motivation reflect crucial boundary conditions that determine the use of conflict‐motivated coping strategies. So far, it has been suggested that people who experience ambivalence are more likely to make food choices against their predominant attitude as a consequence of more effortful coping (e.g., Finkhäuser et al., 2025; Pauer et al., 2022); and people who experience dissonance about eating meat tend to cope with these conflicts in a way that allows them to maintain their commitments (e.g., Bastian & Loughnan, 2017; Loughnan et al., 2010; Rothgerber, 2014). Our framework accounts for these presumably opposing effects of meat‐related conflict by proposing that people are more open in how they cope with ambivalence as compared with when they cope with dissonance. This is because people have a lowered capability for behaviour change after having committed to a meat‐based food choice, and dissonance (compared with ambivalence) motivates people to maintain the chosen course of action (Buttlar, Pauer, & van Harreveld, 2025; Harmon‐Jones et al., 2009). Nonetheless, people may overcome these tendencies and engage in different routes of coping with conflict if its strength increases and the conflict frequently recurs, both of which can increase people's motivation to engage in more systematic problem‐focused coping. Such coping facilitates decision‐making against the predominant attitude or behavioural commitment, eventually rendering behaviour change possible. However, even if people are capable and motivated to cope more systematically with their conflicts, external factors may affect the perceived opportunity for behaviour change and thereby subvert people's inclination to do so. Future research should determine how people incorporate these factors into their decision‐making, especially in regard to the sequence in which they are taken into account.

A promising avenue for future research is thus to test the mechanisms of how conflict strength and recurrence influence people's capability and motivation to regulate the different kinds of conflict and the factors contributing to these processes, in particular, attitude moralization, and valence. In fact, capability and motivation as internal factors and opportunity as an external factor may affect each other (Bryant et al., 2023; Michie et al., 2011), such as when a heightened motivation leads people to cope so that they learn new recipes, and thereby increase their capability for behaviour change. Investigating this interplay in omnivores and veg*ans provides a fruitful opportunity, as these dietary groups differ systematically in the nature and structure of their meat‐related conflicts. For this purpose, within‐person studies examining how dietary groups differentially experience and cope with meat‐related conflicts are essential for illuminating how these processes unfold over time (Brandt & Morgan, 2022; Hofmann & Grigoryan, 2023; Hopwood et al., 2022). For instance, the effect of recurring conflict on behavioural change could possibly follow a non‐linear trajectory when the experience of conflict becomes chronic over prolonged time periods and eventually motivates people to revert to emotion‐focused coping (Pauer et al., 2026). Such intertemporal insights on how meat‐related conflict evolves over time could yield important boundary conditions of how people either fail or succeed in transitioning towards plant‐based diets.

### Practical implications

Going beyond previous reviews on meat‐related conflict (e.g., Bastian & Loughnan, 2017; Rothgerber, 2020; Rothgerber & Rosenfeld, 2021), we outline factors that determine how different dietary groups cope with meat‐related conflicts. Practitioners who aim to change behaviour should not only try to elicit conflict and counteract emotion‐focused or biased problem‐focused coping (e.g., Buttlar et al., 2021); instead, they may aim to increase its strength and recurrence to promote people's motivation to engage in systematic problem‐focused coping. Conflict strength in omnivores can, for instance, be promoted by appealing to moralized (e.g., animal welfare issues) instead of personal (e.g., health risks) attitude components underlying the conflicts. Conflict recurrence can, for instance, be facilitated by making omnivores aware of and counteracting conflict avoidance strategies. By increasing people's motivation this way, they would be enabled to engage in less biased coping to resolve the inconsistency underlying their conflicts, but cope in a way that facilitates optimal decision‐making. Thereby, we not only explain why some interventions (e.g., communicating environmental issues) seem to be more effective than others (e.g., communicating health issues; see, for example, Grundy et al., 2022) but also give a theoretical basis for crafting them by showing how to leverage cognitive conflicts to motivate behaviour change.

Whereas previous research has outlined the consequences of cognitive conflict in omnivores (Gradidge et al., 2021; van Gent et al., 2024), the current review highlights that it is important to consider how veg*ans experience conflict. In fact, cognitive conflict seems to play a crucial role in why many veg*ans violate their diets or revert to an omnivorous diet altogether (Buttlar et al., 2023; Buttlar, Pauer, Scherrer, et al., 2025; Rosenfeld & Tomiyama, 2019). Crucially, some of the passive avoidance strategies that help omnivores to avert conflict can make it more likely that veg*ans experience conflict, increasing the risk of dietary backsliding. Our review thereby suggests that it is particularly important to prevent veg*ans' attitudes from being pulled away from moralization. As such, we provide a comprehensive understanding of how and when both omnivores and veg*ans experience and resolve cognitive conflict. This could help practitioners understand and support people in changing (morally) troublesome behaviours, like meat consumption, and encourage the maintenance of healthy, ethical, and sustainable lifestyles. This may include, for example, supporting veg*ans to maintain newfound diets by addressing health and social obstacles that typically give rise to meat‐related conflict.

## CONCLUSION

Our review delineates how dietary groups are differentially affected by meat‐related conflicts, the timing and triggers of these conflicts, and the downstream consequences and their boundary conditions. Thereby, we challenge established notions in the literature on cognitive conflict and raise several novel research questions to disentangle the meat paradox and deepen our understanding of conflict and its role in behaviour change and maintenance.

## AUTHOR CONTRIBUTIONS

**Benjamin Buttlar:** Conceptualization; methodology; supervision; visualization; project administration; writing – original draft; writing – review and editing; investigation. **Shiva Pauer:** Conceptualization; methodology; writing – review and editing; investigation.

## Data Availability

There is no data attached to this review.
